# Occupational exposure to ionizing radiation in medical staff: trends during the 2009–2019 period in a multicentric study

**DOI:** 10.1007/s00330-023-09541-z

**Published:** 2023-03-17

**Authors:** Clémence Baudin, Blandine Vacquier, Guillemette Thin, Lamine Chenene, Joël Guersen, Isabelle Partarrieu, Martine Louet, Hubert Ducou Le Pointe, Stéphanie Mora, Catherine Verdun-Esquer, Juliette Feuardent, Frédéric Rousseau, Hervé Roy, Lynda Bensefa-Colas, Louis Boyer, Marie-Odile Bernier

**Affiliations:** 1 Laboratoire d’Epidémiologie, Institut de Radioprotection et de Sûreté Nucléaire (IRSN), 31, avenue de la Division Leclerc, F-92260 Fontenay-aux-Roses, France; 2 Service Santé Travail Environnement, CHU de Bordeaux, Bordeaux, France; 3 Service de médecine du travail, Hôpital Cochin, AP-HP, Paris, France; 4 Service central de santé au travail, AP-HP, Paris, France; 5 CHU de Clermont-Ferrand, Clermont-Ferrand, France; 6 Service de médecine du travail, Hôpital Pitié Salpêtrière, AP-HP, Paris, France; 7 Bureau d’Analyse et de Suivi des Expositions Professionnelles, Institut de radioprotection et de Sûreté Nucléaire, Fontenay-aux-Roses, France

**Keywords:** Radiation, ionizing, Occupational exposure, Medical staff

## Abstract

**Objectives:**

Health workers exposed to ionizing radiation account for  + 50% of workers exposed to man-made radiation in France. Over the last decade, the use of radiation in medicine has increased due to the introduction of new practices. The EXposition des Professionnels de santE aux RayonnemenTs ioniSants study aims to evaluate and characterize the trends in radiation exposure of health workers in France between 2009 and 2019.

**Methods:**

This retrospective study includes all health workers with at least one dosimetric record in the system for occupational dosimetry registration (Système d’information de la surveillance de l’exposition aux rayonnements ionisants) database for each of the years 2009, 2014, and 2019, in the hospitals included in the study. Individual external doses and socio-professional data were collected. Statistical analyses include descriptions, graphs, and logistic regressions.

**Results:**

A total of 1457 workers were included (mean age: 39.8 years, 59% women). The average exposure significantly decreased between 2009 and 2019 (−0.008 mSv/year, *p* < 0.05). There were large discrepancies in trends according to professions, departments, hospitals, and gender. Over the 10-year study period, radiologic technologists and physicians were the most exposed (0.15 mSv (95%CI 0.14–0.16) and 0.13 mSv (0.06–0.21), respectively), but their exposure tended to decrease. Workers in nuclear medicine departments had the highest radiation exposure (0.36 mSv (0.33–0.39)), which remained stable over time. Thirty-eight percent of recorded doses were nonzero in 2009, decreasing to 20% in 2019.

**Conclusions:**

This study allowed to identify physicians and radiologic technologists in nuclear medicine departments as the most exposed medical workers in France, and to show an overall decrease trend in radiation exposure. This should be instructive for radiation monitoring and safety of exposed medical workers.

**Key Points:**

*• Radiation exposure of healthcare workers in most medical departments has steadily decreased between 2009 and 2019 in several French hospitals.*

*• The number of zero doses consistently increased during the study period.*

*• Workers in nuclear medicine departments are the most exposed, especially radiologic technologists and physicians.*

**Supplementary Information:**

The online version contains supplementary material available at 10.1007/s00330-023-09541-z.

## Introduction

Medical staff can be involved in X-ray imaging or other techniques, resulting in exposure to ionizing radiation (referred to as *radiation*), which makes them among the most frequently exposed workers [1]. The International Commission on Radiological Protection (ICRP) recommends limits on occupational radiation exposure: 20 mSv per year effective dose averaged over defined 5-year periods, not exceeding 50 mSv in a single year [2].

Occupational exposure has the potential to cause acute effects at high doses [1, 3]. However, such a relationship is not clear in recent medical worker cohorts who are exposed to chronic low doses [4]. Furthermore, the use of radiation in medicine has expanded rapidly over the past 20 years due to the introduction of new diagnostic and therapeutic practices [5–8]. This led to a greater radiation exposure of the medical staff in certain departments [9]. The health effect study of exposure to low doses in health workers requires firstly its precise characterization according to the profession and the medical department, as substantial differences may be expected—which has not been done on a large scale and in a dynamic way over time in France. Only few international studies have detailed the trends of radiation exposure in the last decades, but this has been carried out only for specific professions such as radiologic technologists and doctors, or mainly in patients [10–12].

While any worker possibly exposed to radiation must wear a whole body dosimeter—an important tool to ensure compliance with regulatory or accepted dose limits—a large amount of individual records of doses (dosimetrics) are available to be analyzed [13]. This data is registered and managed in France by the Système d’information de la surveillance de l’exposition aux rayonnements ionisants (SISERI), which records the radiation exposure for every workers likely to be exposed to more than 1 mSv in France [14]. In 2005, SISERI was implemented by law, requiring any company involved with occupational exposure to complete this database with dosimetric information for each of their workers.

The EXposition des Professionnels de santE aux RayonnemenTs ioniSants (EXPERTS) study was set up in 2020 with two objectives: to evaluate and characterize radiation exposure of health workers in France from 2009 to 2019 and to assess the perception of these workers regarding the risks involved after radiation exposure as well as their use of radiation protection tools to protect themselves from radiation. The present paper aims to show the trends in radiation exposure of healthcare workers in France over the last 10 years, considering age, gender, profession, and medical department.

## Methods

### Study population: inclusion and exclusion criteria

This retrospective cohort study included all healthcare workers (≥18 years of age) who had at least one dosimetric recording in the SISERI database for each of the years 2009, 2014, and 2019, in one of the three participating sites (each site consisting of three hospitals, leading to a total of nine hospitals in metropolitan France).

All workers who had quit their jobs, resulting in the end of their monitoring, or who had moved to another hospital between 2009 and 2019 were excluded from the study.

### Data collection

Firstly, socio-professional data (year of birth, medical department, profession) and data on external exposure from dosimeter monitoring for all the healthcare workers meeting inclusion criteria were extracted from the SISERI database. Secondly, professions and medical departments of each worker were checked with available information from the database provided by the software dedicated to hospital occupational health services (the CHIMED^©^ software) in collaboration with the occupational medicine physicians of each hospital.

Personal dose equivalent Hp(10) for each worker was provided for every year between 2009 and 2019, and was estimated from badges (radio-photoluminescence dosimeters) which are, in France, worn at chest level under the lead apron, and considered here as the whole body exposure. All dose values were considered in this work, including doses equal to 0 (“zero dose”). However, no information was provided on the interpretation of the latter; i.e., either the worker was not exposed at all, or he/she was minimally exposed with an exposure value below the detection limit of the dosimeter. The threshold for recording Hp(10) dosimetry data by dosimeters is set at 0.05 mSv, meaning that lower exposures are considered as zero [15].

### Statistical analyses

All departments with less than 20 workers were grouped into the “Other” category.

Both the entire recorded doses for the EXPERTS worker population (referred to as *whole population*) and the nonzero dose population (referred to as *non0 subpopulation*) were considered in this study.

Overall means (OM) of personal dose equivalent Hp(10) over the period 2009–2019 and linear trends in annual mean doses (AMD), i.e., the mean of personal dose equivalent Hp(10) by year, between 2009 and 2019 were calculated on both the whole population and the non0 subpopulation, and then by medical department, profession, gender, and quartile of age at inclusion, respectively.

Then, in the whole population, logistic regressions were used with the recording of a nonzero dose (“yes”/ “no”) as an outcome variable, and the year of exposure as a quantitative factor of interest (M0 model). This was possible after first testing the assumption of linearity between the recording of a nonzero dose and the year of exposure. Potential modifying effects of medical department, profession, gender, and age at inclusion were assessed by including an interaction term between the year of exposure and these covariates consecutively in the M0 model.

Next, in the whole population, the temporal variations of AMD between 2009 and 2019 as well as the temporal variations in nonzero dose percentage were screened by medical department, profession, and quartile of age at inclusion using graphs (for a better reading of the graphs, only the most important group or the most exposed professions/medical departments, or for which the variation was the most important were selected to appear in the graphs). The relationship between AMD and the number of nonzero doses was assessed using a linear regression.

Finally, to assess the trends in the number of “extreme doses,” we counted for each year between 2009 and 2019 the number of workers with doses greater than or equal to the dose threshold corresponding in 2009 to the P75 of the population.

Data were entered and analyzed using the SAS software V. 9.4 (SAS Institute). Statistical significance was defined by *p* <  0.05.

## Results

### Data description

The present study included 1457 health workers, with a mean age at inclusion of 39.8 years old (min = 19; max = 64) and a majority of women (59%). The most frequent professions were nurses (30%), radiologic technologists (28%), and physicians (27%). The medical departments of conventional radiology and surgery (including neurosurgery, orthodontics, thoracotomy, urology, vascular and visceral) represented nearly 50% of the EXPERTS study population. If we look at the distribution by medical activities, more than 40% of the population of EXPERTS workers are involved in interventional radiology (Table S2).

The OM over the 2009–2019 period for the whole population was low (OM = 0.10 mSv, 95%CI 0.10–0.11, max = 24.74, Table 1), with a mean of the cumulative personal dose equivalent Hp(10) over the 2009–2019 period of 1.12 mSv (95%CI 0.95–1.30), max = 76.43 (Table S1). There were only a few personal dose equivalent Hp(10) above 5 mSv during the study period, with only one above the 20 mSv dose limit in 2014 in a surgery department. However, there were only about 27% of nonzero doses among all EXPERTS workers between 2009 and 2019. For the non0 subpopulation, the OM was almost four times higher than for the whole population (OM = 0.39 mSv, 95%CI 0.37–0.42, Table 1).

**Table 1:** Description of radiation exposure in the EXPERTS study, according to socio-professional covariates

	No. of workers	OM (mSv) in the whole population over the 2009–2019 period					OM (mSv) in the non0 subpopulation over the 2009–2019 period				
		No. of doses	Mean (95%CI)	P75	Max	*p*^a^	No.^b^ (%) of nonzero doses	Mean (95%CI)	P75	Max	*p*^a^
All workers	1457	15,779	0.10 (0.10–0.11)	0.05	24.74		4193 (27)	0.39 (0.37–0.42)	0.40	24.74	
By medical department						** < 0.001**					** < 0.001**
Nuclear medicine	112	1221	0.36 (0.33–0.39)	0.59	2.82		647 (53)	0.67 (0.63–0.72)	1.03	2.82	
Interventional radiology	95	1043	0.20 (0.17–0.23)	0.23	9.05		502 (48)	0.42 (0.35–0.48)	0.43	9.05	
Cardiology	113	1233	0.11 (0.09–0.13)	0.06	5.65		361 (29)	0.37 (0.30–0.44)	0.36	5.65	
Operating room	58	624	0.08 (0.07–0.09)	0.10	1.15		207 (33)	0.24 (0.22–0.27)	0.32	1.15	
Surgery	370	3991	0.08 (0.06–0.10)	0.00	24.74		759 (19)	0.41 (0.30–0.52)	0.25	24.74	
Anesthetic and intensive care	172	1836	0.06 (0.05–0.07)	0.00	1.95		424 (23)	0.27 (0.24–0.29)	0.35	1.95	
Conventional radiology	335	3646	0.06 (0.06–0.07)	0.00	8.00		795 (22)	0.29 (0.26–0.33)	0.28	8.00	
Radiotherapy	59	641	0.05 (0.02–0.08)	0.00	8.97		141 (22)	0.23 (0.09–0.37)	0.16	8.97	
Pediatrics	26	283	0.04 (0.01–0.07)	0.00	3.71		33 (12)	0.35 (0.11–0.58)	0.28	3.71	
Other*	85	911	0.07 (0.05–0.10)	0.00	5.90		199 (22)	0.34 (0.22–0.46)	0.23	5.90	
Unknown	32	350	0.12 (0.09–0.14)	0.12	2.60		125 (36)	0.32 (0.26–0.38)	0.44	2.60	
By professions						** < 0.001**					** < 0.001**
Radiologic technologist	414	4514	0.15 (0.14–0.16)	0.10	8.97		1551 (34)	0.44 (0.42–0.47)	0.65	8.97	
Dentist	403	454	0.13 (0.06–0.21)	0.10	17.50		190 (42)	0.32 (0.14–0.50)	0.30	17.50	
Physician	42	4351	0.13 (0.11–0.15)	0.05	24.74		1119 (26)	0.49 (0.41–0.58)	0.35	24.74	
Pharmacist	6	64	0.08 (0.03–0.12)	0.07	0.90		18 (28)	0.27 (0.15–0.40)	0.40	0.90	
Caregiver	92	1002	0.05 (0.03–0.07)	0.00	8.00		156 (16)	0.34 (0.20–0.47)	0.30	8.00	
Nurse	437	4709	0.05 (0.05–0.05)	0.00	2.03		1002 (21)	0.23 (0.22–0.25)	0.30	2.03	
Engineer	11	117	0.04 (0.02–0.06)	0.00	0.64		25 (21)	0.19 (0.11–0.26)	0.23	0.64	
Technician	38	414	0.04 (0.02–0.05)	0.00	1.36		64 (15)	0.23 (0.16–0.29)	0.31	1.36	
Unknown	14	154	0.16 (0.11–0.22)	0.18	2.60		68 (44)	0.37 (0.28–0.47)	0.48	2.60	
By gender						** < 0.001**					** < 0.001**
Men	601	6526	0.13 (0.11–0.14)	0.06	24.74		1810 (28)	0.46 (0.41–0.52)	0.40	24.74	
Women	856	9253	0.09 (0.08–0.09)	0.05	17.50		2383 (26)	0.34 (0.31–0.36)	0.40	17.50	
By age at inclusion						**0.41**					**0.78**
1st quartile (≤ 33 y.o.)^b^	381	4076	0.11 (0.10–0.12)	0.06	8.97		1183 (29)	0.38 (0.35–0.41)	0.44	8.97	
2nd quartile (33–40 y.o.)	368	3991	0.10 (0.09–0.12)	0.05	17.50		1036 (26)	0.40 (0.34–0.46)	0.44	17.50	
3rd quartile (40–47 y.o.)	353	3843	0.09 (0.08–0.11)	0.00	8.91		955 (25)	0.38 (0.33–0.42)	0.40	8.91	
4th quartile (> 47 y.o.)	354	3858	0.11 (0.09–0.13)	0.05	24.74		1015 (26)	0.41 (0.34–0.48)	0.35	24.74	

^a^*p* value for the *F*-test following an ANOVA for comparisons of means between covariate classes

^b^Raw percentage as compared to the total number of doses

^*^Other: gynecology, physical rehabilitation, ENT, pneumology, anatomopathology, pharmacy, emergency medical service (EMS), ambulatory, administrative, radiation protection, endocrinology, rheumatology, neurology, laboratory, maintenance-logistics

*y.o.*, years old; *SD*, standard deviation; *P75*, 75th percentile

Bold values are statistically significant results

The most exposed workers are shown to be mainly men, radiologic technologists, dentists, and physicians, working in nuclear medicine, interventional radiology, and cardiology departments. Age was not significantly associated with radiation exposure as OM were similar across quartiles of age (*p* > 0.05).

### Trends in radiation exposure

For the whole population, the AMD experienced a significant decreased trend of  −0.008 (95%CI  −0.010 to  −0.005) mSv/year between 2009 and 2019, close to linearity (*R*^2^ = 0.85) (Table 2), or a 58% decrease between the means in 2009 and 2019 (Table S1). Nevertheless, large discrepancies can be noticed due to the medical department, the profession, or the gender. Physicians, pharmacists, and radiologic technologists were the workers for which AMD decreased the most between 2009 and 2019, as well as pediatrics, interventional radiology, and cardiology departments. Also, the previously mentioned decrease was more pronounced for men than for women. On the other hand, a nonsignificant increase trend of AMD between 2009 and 2019 was found only for the dentists, or the workers from the nuclear medicine and radiotherapy departments. Discrepancies in trends of AMD (all pointing towards a decrease) were less prominent by age quartile, although the trend towards a decrease in the 4th age quartile at inclusion was the strongest (−0.010 mSv/year (95%CI  −0.016 to  −0.004)).

**Table 2:** Trends in AMD and OR for the recording of a nonzero dose in the EXPERTS study over the 2009–2019 period, according to different socio-professional covariates

	Whole population					Non0 subpopulation	
	*N*	*β*^a^ (95%CI)	*R*^2^	OR^b^ (95%CI)	*p*_interaction_^c^	*β*^a^ (95%CI)	*R*^2^
All workers	1457	−0.008 (−0.010 to −0.005)	0.87	0.90 (0.89–0.91)		0.028 (−0.009 to 0.065)	0.22
By medical department					< 10^−3^		
Pediatrics	26	** −0.014 (−0.022 to −0.006)**	0.62	**0.74 (0.64–0.85)**		** −0.068 (−0.129 to −0.007)**	0.56
Interventional radiology	95	** −0.019 (−0.032 to −0.006)**	0.56	**0.82 (0.78–0.85)**		0.002 (−0.018 to 0.022)	0.01
Cardiology	113	** −0.017 (−0.022 to −0.011)**	0.85	**0.87 (0.83–0.90)**		** −0.024 (−0.038 to −0.009)**	0.60
Operating room	58	** −0.012 (−0.018 to −0.006)**	0.71	**0.82 (0.78–0.87)**		** −0.013 (−0.023 to −0.003)**	0.47
Anesthetic and intensive care	172	** −0.009 (−0.015 to −0.003)**	0.56	**0.92 (0.88–0.95)**		** − 0.022 (− 0.035 to −0.009)**	0.63
Conventional radiology	335	** −0.008 (−0.011 to −0.006)**	0.88	**0.94 (0.91–0.96)**		** −0.023 (−0.037 to −0.009)**	0.60
Surgery	370	−0.003 (−0.010 to 0.004)	0.11	**0.88 (0.86–0.90)**		0.035 (−0.006 to 0.076)	0.30
Radiotherapy	59	0.003 (−0.014 to 0.008)	0.04	**0.93 (0.87–0.99)**		−0.002 (−0.045 to 0.040)	0.00
Nuclear medicine	112	0.005 (−0.002 to 0.011)	0.24	1.00 (0.96**–**1.03)		0.008 (−0.005 to 0.021)	0.17
Other*	85	** −0.006 (−0.009 to −0.003)**	0.67	**0.91 (0.87–0.96)**		−0.004 (−0.028 to 0.020)	0.02
Unknown	32	** −0.022 (−0.031 to −0.013)**	0.77	**0.80 (0.74–0.86)**		** −0.034 (−0.054 to −0.014)**	0.62
By professions					< 10^−3^		
Physician	403	** −0.009 (−0.014 to −0.004)**	0.67	**0.91 (0.89–0.93)**		−0.002 (−0.027 to 0.023)	0.00
Pharmacist	6	−0.009 (−0.021 to 0.004)	0.21	0.92 (0.77**–**1.09)		−0.003 (−0.039 to 0.034)	0.00
Radiologic technologist	414	** −0.008 (−0.011 to −0.005)**	0.82	**0.92 (0.90–0.94)**		0.00 (−0.009 to 0.010)	0.00
Caregiver	92	−0.007 (−0.013 to 0.000)	0.38	**0.93 (0.88–0.98)**		−0.021 (−0.071 to 0.029)	0.09
Nurse	437	** −0.007 (−0.009 to −0.004)**	0.83	**0.88 (0.86–0.90)**		** −0.012 (−0.024 to −0.001)**	0.40
Technician	38	−0.005 (−0.011 to 0.001)	0.32	**0.81 (0.74–0.89)**		0.005 (−0.016 to 0.025)	0.03
Engineer	11	−0.004 (−0.011 to 0.004)	0.13	0.94 (0.82**–**1.08)		−0.009 (−0.036 to 0.019)	0.06
Dentist	42	0.002 (−0.024 to 0.028)	0.00	**0.87 (0.82–0.93)**		0.048 (−0.071 to 0.167)	0.08
Unknown	14	** −0.030 (−0.049 to −0.010)**	0.57	**0.83 (0.74–0.92)**		** −0.042 (−0.070 to −0.014)**	0.56
By gender					0.96		
Men	601	** −0.010 (−0.015 to −0.006)**	0.76	**0.90 (0.89–0.92)**		−0.005 (−0.024 to 0.013)	0.04
Women	856	** −0.006 (−0.008 to −0.004)**	0.82	**0.90 (0.89–0.92)**		0.003 (−0.008 to 0.014)	0.04
By age at inclusion					0.04		
1st quartile (≤ 33 y.o.)	381	** −0.008 (−0.010 to −0.006)**	0.88	**0.88 (0.86–0.90)**		0.008 (−0.002 to 0.017)	0.28
2nd quartile (33–40 y.o.)	368	−0.004 (−0.009 to 0.001)	0.24	**0.91 (0.89–0.93)**		0.018 (−0.007 to 0.044)	0.22
3rd quartile (40–47 y.o.)	353	** −0.009 (−0.011 to −0.006)**	0.87	**0.91 (0.89–0.93)**		−0.011 (−0.025 to 0.003)	0.27
4th quartile (> 47 y.o.)	354	** − 0.010 (−0.016 to −0.004)**	0.61	**0.92 (0.90–0.94)**		−0.018 (−0.036 to 0.001)	0.33

Data in bold are statistically significant results

^a^Slope of the linear trend line from the AMD of Hp(10) doses

^b^Odds ratio (95% confidence interval) for the recording of a nonzero dose in relation to a 1-year increase of exposure

^c^*p* value for the interaction test between the year of exposure and the socio-professional covariates

^*^Other: gynecology, physical rehabilitation, ENT, pneumology, anatomopathology, pharmacy, emergency medical service (EMS), ambulatory, administrative, radiation protection, endocrinology, rheumatology, neurology, laboratory, maintenance logistics

Simultaneously, a 1-year increase in radiation exposure was significantly associated with a 10% decrease in the odds of recording a nonzero dose for the whole population (OR = 0.90, 95%CI 0.89–0.91, Table 2 and Table S2). While gender did not modify this relationship, there were discrepancies between medical departments, medical activity, professions, and quartiles of age: the annual decrease in the number of nonzero doses was more pronounced in technicians, dentists, and nurses, as well as in pediatrics, interventional radiology departments, and operating rooms, and also in half of the youngest workers. The hypothesis that OR of recording a nonzero dose decreases linearly with the year of exposure was verified using the data shown in Figure S1.

**Fig. 1:**
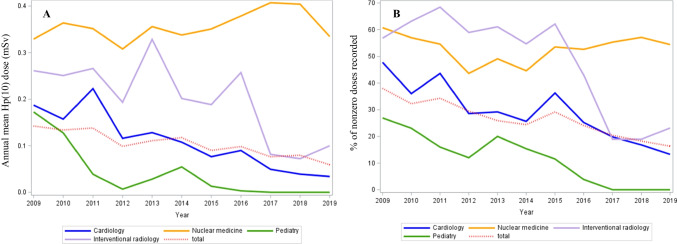
Trends in AMD (**A**) and in nonzero dose percentage (**B**) over the 2009–2019 period according to the department

**Fig. 2:**
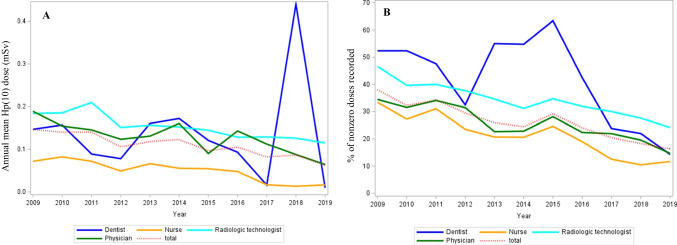
Trends in AMD (**A**) and in nonzero dose percentage (**B**) over the 2009–2019 period according to the profession

**Fig. 3:**
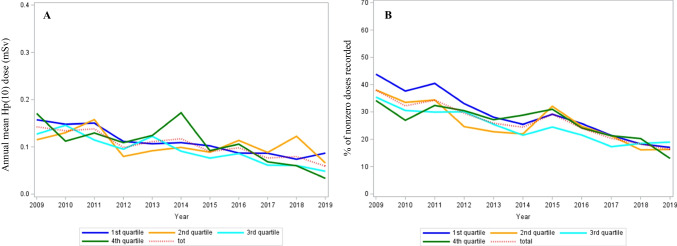
Trends in AMD (**A**) and in nonzero dose percentage (**B**) over the 2009–2019 period according to the age at inclusion

Figures 1, 2, and 3 were made possible to complete the results from the previous tables and to observe the variations of the annual means and the nonzero dose percentage, respectively. The exposure of nuclear medicine workers, which was the highest among all the departments studied, remained roughly stable over time with an AMD between 0.3 and 0.4 mSv. Since 2016, the AMD subsided to zero in pediatrics departments and decreased substantially in interventional radiology departments (Fig. 1A). Regarding professions, all of them experienced a decrease in AMD during the study period. It should be noted that a peak in exposure was observed for dentists in 2018, due only to one dentist whose annual exposure amounted to 17.5 mSv that year, while all other dentists had an exposure close to 0 in the same time frame (Fig. 2A). As for gender, men had a significantly higher average exposure than women until 2018, but their respective exposure decreased until both genders were close to 0.06 mSv in 2019 (graph not shown). Radiation exposure decreased over time in all four age quartiles in a similar manner (Fig. 3A).

The variations in AMD and in the nonzero dose percentage by profession, by medical department, or by quartile of age at inclusion appeared roughly symmetrical (Figs. 1B, 2B, and 3B). The nonzero dose percentage in the nuclear medicine department was approximately stable over time (around 55%), while the percentage in cardiology decreased steadily. Since 2016, approximately 100% of the doses were zero in pediatrics departments, whereas the nonzero dose percentage has decreased considerably since 2016 in interventional radiology departments (Fig. 1B). The nonzero dose percentage has evolved in the same way for men and women during the 10-year period. The decrease in percentage was more pronounced until 2014 for the first quartile of age, although the proportion of nonzero values was about the same for all ages in 2019 (Fig. 3B). Figure S2 shows a statistically significant linear relationship between AMDs and the number of nonzero doses (*β* = 0.0002; *p* < 10^−3^).

**Fig. 4:**
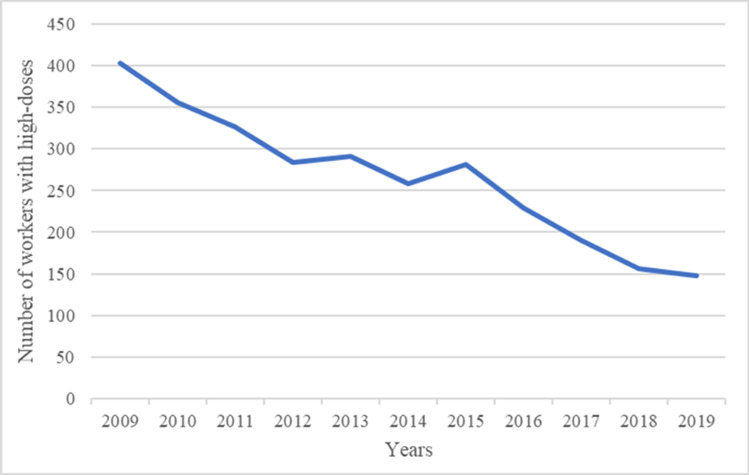
Trend in the number of doses equal or greater than the dose in 2009 corresponding to the 75th percentile of the study population (0.10 mSv)

Finally, the number of workers with doses greater than or equal to the dose threshold corresponding in 2009 to the P75 of the population significantly decreased from 2009 to 2019 (Fig. 4).

## Discussion

Medical staff represents the largest group of radiation-exposed workers in the world [1]. Moreover, the use of medical radiation is increasing, particularly through the use of new procedures [5, 6]. This paper offers the possibility to study the trends in occupational exposure over the past 10 years in medical workers in France.

Our analyses showed that monitored healthcare workers have a low OM (0.10 mSv (95%CI 0.10–0.11) over 2009–2019), and only one annual personal dose equivalent Hp(10) was recorded above the regulatory limit (> 20 mSv) in 2014 [2]. The doses decreased regularly over the study period, but discrepancies were found both in OM and in AMD trends according to professions, medical departments, medical activity, and gender. To our knowledge, only two recent similar studies have been carried out on occupational exposure of medical workers, but the methodology was different and the level of exposure was higher than in our study, thus preventing comparisons [11, 16]. Also, a United Nations Scientific Committee on the Effects of Atomic Radiation (UNSCEAR) report includes an estimate of the average annual effective doses received by healthcare workers worldwide, and an analysis of temporal trends in occupational exposure, based on a review of the literature and surveys [17]. The average annual effective dose from all medical uses of radiation was 0.09 mSv in France for the 2010–2014 period (very similar to that of our workers), which makes it one of the countries in which medical workers are the least exposed.

The decrease in exposure observed in this study seems supported by a decrease of the nonzero dose percentage. This could be due primarily to exposure falling below the dosimeter detection limit (0.05 mSv) rather than a cessation of radiation exposure, as workers who left their jobs during the study period were excluded. The assumption that workers no longer wear their dosimeters during the study period is unlikely because radiation protection awareness is a growing concern. Substantial technological improvements and efforts are made to optimize the dose delivered per procedure [9], which may explain the fall in exposure below the dosimeter detection limit. Three principles (i.e., justification, optimization, and limitation) have been established by the ICRP to protect workers and patients from radiation health risks, also stating “the ALARA principle” (As Low As Reasonably Achievable) [18].

Unfortunately, our results may be underestimated because they are contingent on the acceptance of the dosimeter by the medical staff, which is not always the case during radiation procedures [19]. Moreover, it was not possible to distinguish the true decrease of doses from a lack of exposure due to change in the work, such as nurses becoming team leaders during the study period. However, if this were the case, we should have observed a greater OR for nonzero dose recording in the last age quartile than in the first age quartile, but the opposite was shown. More developed causality analyses taking into account various latent variables would be needed to further knowledge on this point. Also, one of the problems was the heterogeneity of medical departments and professions in the different hospitals. However, medical departments have been harmonized using the information recorded in the CHIMED^©^ software, which notably increased the quality of the present study. Indeed, the latter software allows occupational health services to create an individual file for each monitored worker, to record health parameters and exposures, and to provide a detailed administrative file allowing to know the professional career of the workers. Even if the medical department and/or the profession of several workers remained unknown, this may have impacted on the results to a lesser extent as they accounted for a small percentage of workers (less than 2%). In addition, an analysis by occupational activity has been introduced in Supplementary materials (Tables S2 and S3), using the same classification as UNSCEAR and the ESOREX platform [20, 21], allowing a larger scale comparison. Nevertheless, we felt it was important to consider the analyses by department in the main analyses, in order to target those where radiation protection action could be taken.

The main strengths of this work include the large sample size of the study, the longitudinal design allowing a follow-up of medical workers over a 10-year period (during which time awareness of the health effects of low doses has emerged), and the use of occupational exposure from a national database, the SISERI database. The latter tool makes it possible to rely on high-quality individual and repeated exposure data. Indeed, in all fields in France, the SISERI system centralizes, verifies, and stores via a secure Internet access all the results of individual measurements of workers’ exposure to radiation, which are regularly transmitted by occupational physicians or Competent Radiological Protection Personnel in accordance with the rules set out in the French Labor Code [13].

The longitudinal design of our study on the trend of individual doses allows us to avoid as much as possible the bias related to the nonuse of dosimeters, which is unfortunately a recurrent problem in dosimetric studies. Indeed, as dosimeter wearing is supposed to be more or less similar over time in the same workers, it is unlikely that the trend in doses is related to the dosimeter wearing evolution. Moreover, the longitudinal design of our work allows to assess the trend of the individual dose over the long term, which is complementary to a study carried out by IRSN with a cross-sectional design. In the latter study, the doses of radiation-exposed workers were analyzed annually for all health workers wearing dosimeters. The 2020 report mentions that the individual mean dose has remained stable from 2015 to 2019 [14].

Furthermore, our study relies on the inclusion of several hospitals with varying throughput (i.e., differences in the number of patients treated and procedures performed, in radiation protection policies, and in the number of workers in the hospital resulting in a different access to radiation protection tools). Moreover, thanks to the combination of the SISERI database with the CHIMED^©^ software, this study benefits from detailed information on the medical departments and professions, leading to analyses for each of them, which is unprecedented in this type of study.

The extraction and analysis of dosimetrics for the 2009–2019 period with regard to the 1457 medical workers included in the study allowed us to identify the most exposed medical workers in France (i.e., physicians and radiologic technologists in nuclear medicine departments) and to assess the trends in radiation exposure over this period, which tend towards a steady decrease. The radiation protection measures to be adopted are based on the identification of risk situations according to the profession and the medical department, and on the optimization of exposures and practices. Each hospital is encouraged to have a radiation protection and safety council in order to allow the follow-up of medical workers’ exposure and health. Long-term epidemiological surveillance of these workers would make it possible to estimate potential long-term effects of radiation exposure at a low dose. Such comparisons could be carried out with data from other countries to deepen the knowledge of radiation protection, especially since a categorization used in this work (see Tables S2 and S3) is similar to that used by UNSCEAR or the ESOREX platform [20, 21].

A large amount of data will be collected from the questionnaire on radiation protection mailed to all the workers included in our study, enabling further analyses. Also, future similar studies can be carried out when sufficient data will be recorded for specific exposures such as finger or eye doses for which only a limited number of data were collected at this time. Finally, further studies would be needed to measure the health effects of this exposure trend.

## Supplementary Information

Below is the link to the electronic supplementary material.

Supplementary file1 (DOCX 152 KB)

## Abbreviations

AMD: Annual mean dose
CI: Confidence interval
EXPERTS: EXposition des Professionnels de santE aux RayonnemenTs ioniSants
ICRP: International Commission on Radiological Protection
mSv: Millisievert
OM: Overall mean of personal dose equivalent Hp(10) over the 2009–2019 period
OR: Odds ratio
P75: 75Th percentile
SD: Standard deviation
SISERI: Système d’information de la surveillance de l’exposition aux rayonnements ionisants

## Declarations

### Guarantor

The scientific guarantor of this publication is Clémence Baudin.

### Statistics and biometry

Clémence Baudin kindly provided statistical advice for this manuscript.

### Methodology

• retrospective

• observational

• multicenter study
