# Supplementary material for: Occupational exposure to ionizing radiation in medical staff: trends during the 2009–2019 period in a multicentric study
Source: Eur Radiol. 2023 Mar 17;33(8):5675–84. doi: 10.1007/s00330-023-09541-z (PMC10326158; doi:10.1007/s00330-023-09541-z)
Supplement: Supplementary file 1 — Supplementary file1 (DOCX 152 KB) [file 330_2023_9541_MOESM1_ESM.docx]

**Supplementary material**


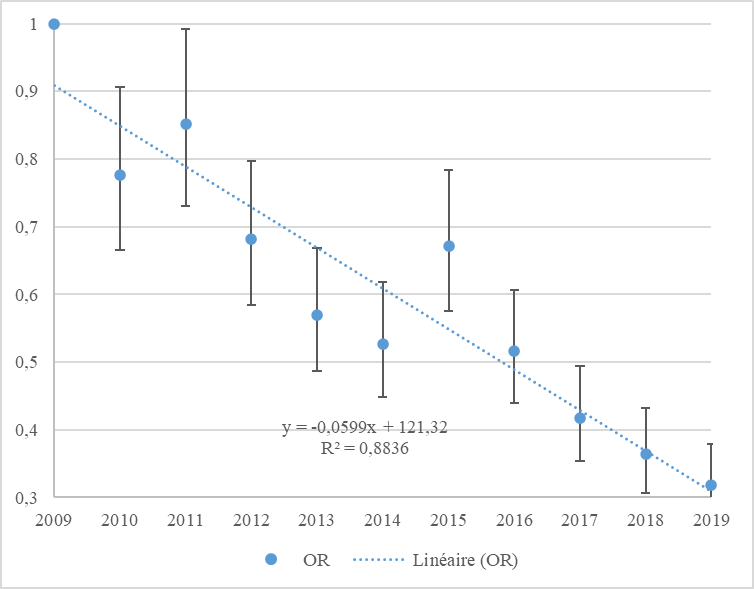


Figure S1: OR for recording a nonzero dose according to the year of exposure


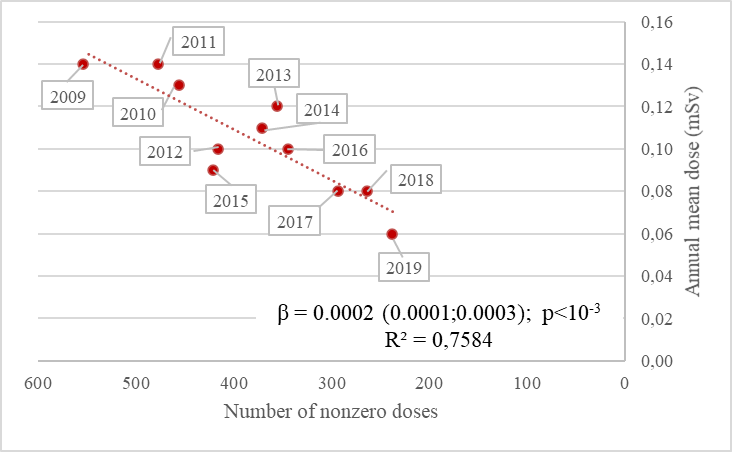


Figure S2: Relationship between AMD and the number of nonzero doses (with the corresponding year in label)

Table S1: Annual dose means for the 2009 to 2019 years (mean (95%CI) in mSv), cumulative dose mean over the 2009-2019 period (mean (±95%CI) in mSv), and relative change between 2009 and 2019 (%)

|  |  | **N** | **AMD 2009** | **AMD 2010** | **AMD 2011** | **AMD 2012** | **AMD 2013** | **AMD 2014** | **AMD 2015** | **AMD 2016** | **AMD 2017** | **AMD 2018** | **AMD 2019** | **Cumulative dose over the 2009-2019 period (mSv)** | **Relative change between 2009-2019 AMD (%)** |
| --- | --- | --- | --- | --- | --- | --- | --- | --- | --- | --- | --- | --- | --- | --- | --- |
| **All workers** | | 1,457 | 0.14 (0.11;0.17) | 0.13 (0.11;0.15) | 0.14 (0.11;0.16) | 0.10 (0.08;0.12) | 0.11 (0.09;0.13) | 0.12 (0.08;0.15) | 0.09 (0.08;0.10) | 0.10 (0.07;0.12) | 0.08 (0.05;0.1) | 0.08 (0.05;0.11) | 0.06 (0.04;0.08) | 1.12 (0.95;1.30) | -58,5 |
|  | **By Medical department** |  |  |  |  |  |  |  |  |  |  |  |  |  |  |
|  | Nuclear medicine | 112 | 0.33 (0.24;0.42) | 0.36 (0.26;0.47) | 0.35 (0.26;0.44) | 0.31 (0.22;0.40) | 0.36 (0.26;0.46) | 0.34 (0.23;0.44) | 0.35 (0.25;0.45) | 0.38 (0.27;0.49) | 0.41 (0.30;0.52) | 0.40 (0.29;0.52) | 0.33 (0.24;0.43) | 3.88 (2.94;4.83) | 1.6 |
|  | Interventional radiology | 95 | 0.26 (0.16;0.37) | 0.25 (0.18;0.33) | 0.27 (0.21;0.32) | 0.19 (0.11;0.28) | 0.33 (0.10;0.55) | 0.20 (0.15;0.25) | 0.19 (0.14;0.24) | 0.26 (0.04;0.48) | 0.08 (0.00;0.16) | 0.07 (0.01;0.13) | 0.10 (0.01;0.19) | 2.20 (1.51;2.88) | -61.7 |
|  | Cardiology | 113 | 0.19 (0.10;0.27) | 0.16 (0.06;0.26) | 0.22 (0.07;0.38) | 0.12 (0.04;0.19) | 0.13 (0.06;0.20) | 0.11 (0.04;0.18) | 0.08 (0.04;0.11) | 0.09 (0.03;0.15) | 0.05 (0.02;0.08) | 0.04 (0.02;0.06) | 0.03 (0.01;0.05) | 1.19 (0.67;1.72) | -81.8 |
|  | Operating room | 58 | 0.11 (0.07;0.15) | 0.12 (0.07;0.17) | 0.11 (0.06;0.16) | 0.11 (0.07;0.16) | 0.14 (0.09;0.20) | 0.12 (0.07;0.17) | 0.06 (0.03;0.10) | 0.07 (0.02;0.11) | 0.02 (0.00;0.03) | 0.02 (0.00;0.03) | 0.02 (0.00;0.04) | 0.87 (0.61;1.13) | -84,0 |
|  | Surgery | 370 | 0.11 (0.01;0.21) | 0.08 (0.05;0.11) | 0.07 (0.04;0.09) | 0.08 (0.03;0.12) | 0.06 (0.03;0.09) | 0.15 (0.01;0.29) | 0.05 (0.02;0.08) | 0.06 (0.02;0.11) | 0.07 (0.00;0.14) | 0.10 (0.00;0.21) | 0.04 (0.00;0.09) | 0.84 (0.36;1.33) | -67.9 |
|  | Anesthetic and intensive care | 172 | 0.08 (0.05;0.11) | 0.14 (0.10;0.19) | 0.11 (0.07;0.15) | 0.03 (0.02;0.05) | 0.06 (0.03;0.09) | 0.04 (0.03;0.06) | 0.08 (0.05;0.11) | 0.07 (0.05;0.09) | 0.02 (0.01;0.03) | 0.02 (0.01;0.03) | 0.02 (0.01;0.03) | 0.65 (0.49;0.81) | -73.5 |
|  | Conventional radiology | 335 | 0.12 (0.08;0.16) | 0.09 (0.06;0.12) | 0.10 (0.05;0.16) | 0.07 (0.05;0.09) | 0.06 (0.04;0.08) | 0.06 (0.04;0.09) | 0.06 (0.03;0.08) | 0.04 (0.02;0.06) | 0.04 (0.03;0.06) | 0.04 (0.03;0.06) | 0.02 (0.01;0.03) | 0.70 (0.51;0.89) | -81.8 |
|  | Radiotherapy | 59 | 0.04 (0.02;0.07) | 0.05 (0.00;0.11) | 0.18 (0.00;0.49) | 0.02 (0.01;0.04) | 0.05 (0.03;0.07) | 0.02 (0.01;0.03) | 0.02 (0.00;0.04) | 0.01 (0.01;0.02) | 0.03 (0.00;0.07) | 0.02 (0.01;0.03) | 0.11 (0.00;0.26) | 0.55 (0.19;0.92) | 141.9 |
|  | Pediatrics | 26 | 0.17 (0.00;0.47) | 0.13 (0.01;0.25) | 0.04 (0.00;0.09) | 0.01 (0.00;0.01) | 0.03 (0.00;0.05) | 0.05 (0.00;0.12) | 0.01 (0.00;0.03) | 0.00 (0.00;0.01) | 0 (0;0) | 0 (0;0) | 0 (0;0) | 0.44 (0.05;0.83) | -100,0 |
|  | Other* | 85 | 0.12 (0.02;0.23) | 0.10 (0.00;0.23) | 0.08 (0.01;0.14) | 0.08 (0.00;0.18) | 0.07 (0.00;0.16) | 0.06 (0.00;0.15) | 0.06 (0.00;0.16) | 0.09 (0.00;0.23) | 0.07 (0.02;0.12) | 0.04 (0.02;0.07) | 0.04 (0.00;0.08) | 0.79 (0.00;1.64) | -66,0 |
|  | *unknown* | 32 | 0.15 (0.07;0.23) | 0.27 (0.08;0.46) | 0.20 (0.08;0.31) | 0.13 (0.05;0.21) | 0.17 (0.07;0.28) | 0.12 (0.02;0.23) | 0.09 (0.03;0.15) | 0.11 (0.05;0.18) | 0.01 (0.00;0.02) | 0.01 (0.00;0.02) | 0.01 (0.00;0.02) | 1.26 (0.70;1.82) | -95.3 |
|  | **By Professions** |  |  |  |  |  |  |  |  |  |  |  |  |  |  |
|  | Radiologic technologist | 414 | 0.18 (0.15;0.22) | 0.19 (0.15;0.22) | 0.21 (0.16;0.26) | 0.15 (0.12;0.18) | 0.16 (0.12;0.19) | 0.15 (0.12;0.19) | 0.14 (0.11;0.18) | 0.13 (0.09;0.16) | 0.13 (0.09;0.16) | 0.13 (0.09;0.16) | 0.12 (0.08;0.15) | 1.67 (1.36;1.97) | -37.5 |
|  | Dentist | 42 | 0.15 (0.08;0.22) | 0.16 (0.08;0.23) | 0.09 (0.05;0.13) | 0.08 (0.02;0.13) | 0.16 (0.08;0.24) | 0.17 (0.07;0.27) | 0.12 (0.08;0.17) | 0.09 (0.04;0.15) | 0.02 (0.01;0.03) | 0.44 (0.00;1.30) | 0.01 (0.00;0.02) | 1.45 (0.49;2.42) | -93.4 |
|  | Physician | 403 | 0.19 (0.09;0.29) | 0.15 (0.10;0.21) | 0.15 (0.09;0.20) | 0.12 (0.07;0.18) | 0.13 (0.07;0.20) | 0.16 (0.03;0.29) | 0.09 (0.05;0.13) | 0.14 (0.07;0.22) | 0.11 (0.04;0.19) | 0.09 (0.03;0.14) | 0.06 (0.01;0.11) | 1.37 (0.85;1.89) | -65.9 |
|  | Pharmacist | 6 | 0.17 (0.00;0.47) | 0.17 (0.00;0.54) | 0.14 (0.00;0.39) | 0.01 (0.00;0.04) | 0.02 (0.00;0.06) | 0.01 (0.00;0.04) | 0.03 (0.00;0.10) | 0.05 (0.00;0.16) | 0.08 (0.00;0.30) | 0.11 (0.00;0.32) | 0.05 (0.00;0.18) | 0.82 (0.00;1.85) | -70.2 |
|  | Caregiver | 92 | 0.10 (0.01;0.19) | 0.05 (0.00;0.09) | 0.13 (0.00;0.31) | 0.03 (0.01;0.05) | 0.04 (0.01;0.07) | 0.08 (0.00;0.22) | 0.03 (0.01;0.04) | 0.04 (0.02;0.06) | 0.03 (0.01;0.06) | 0.03 (0.01;0.05) | 0.03 (0.01;0.05) | 0.57 (0.29;0.86) | -72.6 |
|  | Nurse | 437 | 0.07 (0.06;0.09) | 0.08 (0.06;0.1à) | 0.07 (0.05;0.09) | 0.05 (0.04;0.06) | 0.07 (0.05;0.08) | 0.06 (0.04;0.07) | 0.05 (0.04;0.07) | 0.05 (0.04;0.06) | 0.02 (0.01;0.02) | 0.01 (0.01;0.02) | 0.02 (0.01;0.02) | 0.53 (0.44;0.62) | -77,0 |
|  | Engineer | 11 | 0.02 (0.00;0.05) | 0.02 (0.00;0.04) | 0.07 (0.00;0.21) | 0.11 (0.00;0.28) | 0.09 (0.00;0.22) | 0.04 (0.00;0.10) | 0.01 (0.00;0.03) | 0.03 (0.00;0.07) | 0.02 (0.00;0.05) | 0.02 (0.00;0.07) | 0.01 (0.00;0.03) | 0.42 (0.00;0.87) | -45.8 |
|  | Technician | 38 | 0.11 (0.05;0.17) | 0.04 (0.01;0.07) | 0.05 (0.01;0.09) | 0.03 (0.00;0.05) | 0.01 (0.00;0.01) | 0.05 (0.00;0.13) | 0.02 (0.00;0.05) | 0.02 (0.00;0.04) | 0.01 (0.00;0.03) | 0.01 (0.00;0.02) | 0.05 (0.00;0.12) | 0.38 (0.17;0.59) | -59.3 |
|  | *unknown* | 14 | 0.19 (0.02;0.36) | 0.46 (0.05;0.86) | 0.27 (0.07;0.47) | 0.13 (0.02;0.23) | 0.23 (0.02;0.44) | 0.12 (0.00;0.26) | 0.16 (0.04;0.28) | 0.21 (0.07;0.35) | 0.02 (0.00;0.04) | 0.02 (0.00;0.04) | 0.02 (0.00;0.04) | 1.81 (0.76;2.86) | -91.3 |
|  | **By gender** |  |  |  |  |  |  |  |  |  |  |  |  |  |  |
|  | Men | 601 | 0.19 (0.12;0.26) | 0.16 (0.12;0.20) | 0.17 (0.12;0.22) | 0.12 (0.08;0.16) | 0.12 (0.09;0.16) | 0.16 (0.08;0.25) | 0.11 (0.08;0.14) | 0.14 (0.09;0.19) | 0.10 (0.05;0.15) | 0.08 (0.05;0.12) | 0.06 (0.03;0.10) | 1.40 (1.03;1.77) | -67.3 |
|  | Women | 856 | 0.11 (0.09;0.13) | 0.11 (0.10;0.13) | 0.12 (0.09;0.14) | 0.08 (0.07;0.10) | 0.10 (0.07;0.13) | 0.08 (0.07;0.10) | 0.08 (0.06;0.09) | 0.07 (0.05;0.09) | 0.06 (0.04;0.08) | 0.08 (0.03;0.12) | 0.06 (0.04;0.08) | 0.93 (0.79;1.08) | -47.9 |
|  | **By age at inclusion** |  |  |  |  |  |  |  |  |  |  |  |  |  |  |
|  | 1^st^ quartile (≤33 y.o.) ^b^ | 381 | 0.16 (0.12;0.20) | 0.15 (0.11;0.18) | 0.15 (0.09;0.21) | 0.11 (0.08;0.14) | 0.11 (0.08;0.14) | 0.11 (0.08;0.14) | 0.10 (0.07;0.13) | 0.09 (0.06;0.12) | 0.09 (0.06;0.12) | 0.07 (0.05;0.10) | 0.09 (0.05;0.12) | 1.18 (0.93;1.43) | -44.7 |
|  | 2^nd^ quartile ]33;40 y.o.] | 368 | 0.12 (0.09;0.14) | 0.13 (0.10;0.16) | 0.16 (0.10;0.21) | 0.08 (0.06;0.10) | 0.09 (0.07;0.12) | 0.10 (0.07;0.13) | 0.09 (0.07;0.11) | 0.11 (0.05;0.18) | 0.09 (0.01;0.16) | 0.12 (0.01;0.23) | 0.07 (0.01;0.12) | 1.13 (0.81;1.45) | -42.7 |
|  | 3^rd^ quartile ]40;47 y.o.] | 353 | 0.13 (0.09;0.16) | 0.15 (0.10;0.20) | 0.11 (0.07;0.16) | 0.10 (0.06;0.13) | 0.12 (0.06;0.18) | 0.09 (0.05;0.14) | 0.08 (0.05;0.11) | 0.09 (0.05;0.13) | 0.06 (0.04;0.09) | 0.06 (0.03;0.09) | 0.05 (0.03;0.07) | 1.01 (0.71;1.32) | -62,0 |
|  | 4^th^ quartile (>47 y.o.) | 354 | 0.17 (0.06;0.28) | 0.11 (0.07;0.15) | 0.13 (0.08;0.18) | 0.11 (0.06;0.16) | 0.12 (0.07;0.18) | 0.17 (0.03;0.31) | 0.09 (0.05;0.13) | 0.11 (0.06;0.15) | 0.07 (0.04;0.10) | 0.06 (0.03;0.09) | 0.03 (0.01;0.05) | 1.17 (0.67;1.66) | -80.4 |

*Table S2: Description of radiation exposure in the EXPERTS study, according to medical activities following the UNSCEAR classification*

|  |  | **N workers** | **OM (mSv) in the whole population over the 2009-2019 period** | | | | | **OM (mSv) in the non0 subpopulation over the 2009-2019 period** | | | | |
| --- | --- | --- | --- | --- | --- | --- | --- | --- | --- | --- | --- | --- |
|  |  |  | **N doses** | **Mean (95% CI)** | **P75** | **Max** | **p^a^** | **N^b^ (%) nonzero doses** | **Mean (95% CI)** | **P75** | **Max** | **p^a^** |
|  | **By Medical activity** |  |  |  |  |  | **<.001** |  |  |  |  | **<.001** |
|  | Nuclear medicine | 112 | 1221 | 0.36 (0.33;0.39) | 0.59 | 2.82 |  | 647 (53) | 0.67 (0.63;0.72) | 1.03 | 2.82 |  |
|  | Interventional radiology | 604 | 6550 | 0.10 (0.09;0.12) | 0.05 | 24.74 |  | 1,655 (25) | 0.40 (0.35;0.46) | 0.35 | 24.74 |  |
|  | Operating room | 58 | 624 | 0.08 (0.07;0.09) | 0.10 | 1.15 |  | 207 (33) | 0.24 (0.22;0.27) | 0.32 | 1.15 |  |
|  | Conventional radiology | 335 | 3646 | 0.06 (0.06;0.07) | 0.00 | 8.00 |  | 795 (22) | 0.29 (0.26;0.33) | 0.28 | 8.00 |  |
|  | Radiotherapy | 59 | 641 | 0.05 (0.02;0.08) | 0.00 | 8.97 |  | 141 (22) | 0.23 (0.09;0.37) | 0.16 | 8.97 |  |
|  | Other* | 85 | 911 | 0.07 (0.05;0.10) | 0.00 | 5.90 |  | 199 (22) | 0.34 (0.22;0.46) | 0.23 | 5.90 |  |
|  | *unknown* | 32 | 350 | 0.12 (0.09;0.14) | 0.12 | 2.60 |  | 125 (36) | 0.32 (0.26;0.38) | 0.44 | 2.60 |  |

*^a^ p-value for the F-test following an ANOVA for comparisons of means between covariate classes; ^b^ raw percentage as compared to the total number of doses; *Other: gynecology, physical rehabilitation, ENT, pneumology, anatomopathology, pharmacy, Emergency Medical Service (EMS), ambulatory, administrative, radiation protection, endocrinology, rheumatology, neurology, laboratory, maintenance-logistics; y.o.: years old; SD: standard deviation; P75: 75^th^ percentile*

*Table S3: Trends in AMD and OR for the recording of a nonzero dose in the EXPERTS study over the 2009-2019 period, according to the medical activity following the UNSCEAR classification*

|  |  | **Whole population** | | | | | **Non0 subpopulation** | | |
| --- | --- | --- | --- | --- | --- | --- | --- | --- | --- |
|  |  | **N** | **β^a^ (95% CI)** | **R²** | **OR^b^ (95% CI)** | **p_interaction_^c^** | **β^a^ (95% CI)** | **R²** |  |
|  | **By Medical activity** |  |  |  |  | <10^-3^ |  |  |  |
|  | Operating room | 58 | **-0.012 (-0.018;-0.006)** | 0.71 | **0.82 (0.78;0.87)** |  | **-0.013 (-0.023;-0.003)** | 0.47 |  |
|  | Interventional radiology | 604 | **-0.009 (-0.013;-0.005)** | 0.71 | **0.87 (0.85;0.88)** |  | 0.012 (-0.01;0.034) | 0.14 |  |
|  | Conventional radiology | 335 | **-0.008 (-0.011;-0.006)** | 0.88 | **0.94 (0.91;0.96)** |  | **-0.023 (-0.037;-0.009)** | 0.60 |  |
|  | Radiotherapy | 59 | 0.003 (-0.014;0.008) | 0.04 | **0.93 (0.87;0.99)** |  | -0.002 (-0.045;0.040) | 0.00 |  |
|  | Nuclear medicine | 112 | 0.005 (-0.002;0.011) | 0.24 | 1.00 (0.96;1.03) |  | 0.008 (-0.005;0.021) | 0.17 |  |
|  | Other* | 85 | **-0.006 (-0.009;-0.003)** | 0.67 | **0.91 (0.87;0.96)** |  | -0.004 (-0.028;0.020) | 0.02 |  |
|  | *unknown* | 32 | **-0.022 (-0.031;-0.013)** | 0.77 | **0.80 (0.74;0.86)** |  | **-0.034 (-0.054;-0.014)** | 0.62 |  |

*^a^ Slope of the linear trend line from the AMD of Hp(10) doses; ^b^ Odds ratio (95% confidence interval) for the recording of a nonzero dose in relation to a 1-year increase of exposure; ^c^ p-value for the interaction test between the year of exposure and the socio-professional covariates; *Other : gynecology, physical rehabilitation, ENT, pneumology, anatomopathology, pharmacy, Emergency Medical Service (EMS), ambulatory, administrative, radiation protection, endocrinology, rheumatology, neurology, laboratory, maintenance-logistics; in bold are statistically significant results*
